# Using immersive virtual reality to remotely examine performance differences between dominant and non-dominant hands

**DOI:** 10.1007/s10055-023-00794-z

**Published:** 2023-05-06

**Authors:** Jack Owen Evans, Krasimira Tsaneva-Atanasova, Gavin Buckingham

**Affiliations:** 1 Department of Public Health and Sport Sciences, Richards Building, Magdalen Road, University of Exeter, Exeter, Devon EX2 4TA UK; 2 Department of Mathematics and Statistics, Living Systems Institute, University of Exeter, Exeter, Devon EX4 4QD UK; 3 EPSRC Hub for Quantitative Modelling in Healthcare, University of Exeter, Exeter, Devon EX4 4QD UK

**Keywords:** Meta Quest, Dominant hand, Non-dominant hand, Assessment, Stroke, Upper limb

## Abstract

Circle drawing may be a useful task to study upper-limb function in patient populations. However, previous studies rely on expensive and bulky robotics to measure performance. For clinics or hospitals with limited budgets and space, this may be unfeasible. Virtual reality (VR) provides a portable and low-cost tool with integrated motion capture. It offers potentially a more feasible medium by which to assess upper-limb motor function. Prior to use with patient populations, it is important to validate and test the capabilities of VR with healthy users. This study examined whether a VR-based circle drawing task, completed remotely using participant’s own devices, could capture differences between movement kinematics of the dominant and non-dominant hands in healthy individuals. Participants (*n* = 47) traced the outline of a circle presented on their VR head-mounted displays with each hand, while the positions of the hand-held controllers were continuously recorded. Although there were no differences observed in the size or roundness of circles drawn with each hand, consistent with prior literature our results did show that the circles drawn with the dominant hand were completed faster than those with the non-dominant hand. This provides preliminary evidence that a VR-based circle drawing task may be a feasible method for detecting subtle differences in function in clinical populations.

**Supplementary Information:**

The online version contains supplementary material available at 10.1007/s10055-023-00794-z.

## Introduction

Stroke survivors often experience a range of impairments to their cognitive, sensory, and motor faculties. Upper limb deficits frequently occur after stroke, with up to 80% of survivors experiencing some type of upper limb impairment (Persson et al. 2012; Lawrence et al. 2001; Jørgensen et al. 2000). Many survivors struggle to carry out activities of daily living and become less active in their social lives (Desrosiers et al. 2003; Jørgensen et al. 1995). The ability to provide an accurate and detailed account of bodily function after stroke is imperative to the rehabilitation process. Indeed, clinical assessments and outcome measures are a key tool in a clinician’s repertoire, providing important information on the presence and severity of impairments and aiding in the construction of a rehabilitation plan (Potter et al. 2011; Sullivan et al. 2011; Jette et al. 2009). Outcome measures also help to streamline care services, provide useful statistics on stroke recovery rates, and provide a comparison of outcomes between trials and treatments (Harrison et al. 2013). As such, outcome measures are fundamental in shaping clinical practice (Sullivan et al. 2013).

Despite their high reliability (Kwakkel et al. 2017; Alt Murphy et al. 2015), many of the most commonly used outcome measures for assessing upper limb function after stroke have certain shortcomings. The scoring systems for many of these tests are subjective and lacking in granularity, involving movement observations which are rated using short ordinal scales (Hobart et al. 2007). Such scoring systems are considered generally less sensitive to change than other measures (Alt Murphy et al. 2015; Catz et al. 1997), whereby the rating system may overlook small but important changes in function. In some cases, severely and mildly affected survivors can reach a plateau at which functional changes cease to be recognised (see Lamers et al. 2013; Platz et al. 2005; Hsueh and Hsieh 2002).

Recently, kinematic-based assessments of function have become more prominent. Quantitative measures can provide data that is potentially more objective, detailed, and more precise than current clinical outcome measures and are typically collected through robotics or motion capture using reaching or pointing tasks. Indeed, such studies have shown promising results. For example, Coderre et al. (2010) implemented a visually guided reaching task to assess function after stroke while recording movements using a robotic arm. In contrast to conventional measures, kinematics were able to identify ‘impaired’ movements even in survivors who are classified as ‘normal’ or ‘near normal’ on the Chedoke-McMaster Stroke Assessment Scale. Furthermore, kinematics have been useful for tracking compensatory movements during reaching tasks (van Kordelaar et al. 2012; Alt Murphy et al. 2011; Subramanian et al. 2010) and can provide information on internal processes such as motor planning (Chen et al. 2021; Alt Murphy et al. 2017; Tan et al. 2012; Zollo et al. 2011). In addition to point-to-point and reaching tasks, circle drawing provides a potentially powerful insight into arm function as this task is simple to administer while yielding a wide range of quantitative metrics. It requires the coordination and synchrony of multiple joints and, as such, may be a useful task with which to study upper limb function in clinical populations. Indeed, previous studies have used circle drawing to evaluate movement performance in stroke survivors with upper limb deficits (Dipietro et al. 2007). Kinematic measures such as path length and movement time have good clinimetric properties (see Schwarz et al. 2019a, b), whilst circle drawing metrics (size and roundness) have been shown to correlate well with existing measures of upper limb function, where larger and rounder circles are associated with higher scores on the upper extremity component of the Fugl-Meyer Assessment (FMA) (Krabben et al. 2011).

The majority of kinematic-based assessment studies, like those mentioned above, tend to rely on expensive and bulky robotics to measure performance. For clinics or hospitals with limited budgets and space, this may be unfeasible or potentially unsafe (Laparidou et al. 2021; Shirota et al. 2019; Mao et al. 2014). Virtual reality (VR) provides an alternative which supports integrated motion capture. With the commercialisation of VR head-mounted displays (HMDs) like the Meta Quest (Meta Platforms Inc., California, USA) VR technology is becoming increasingly portable, affordable, and widespread. Furthermore, the integrated motion capture in such devices is often accurate, low-latency, and simple to use (Abdlkarim et al. 2022; Holzwarth et al. 2021; Eger Passos and Jung 2020; Voigt-Antons et al. 2020; Borrego et al. 2018; Niehorster et al. 2017). As such, VR may be a useful and more feasible tool with which to assess functional performance in patient populations. Prior to conducting work with vulnerable populations, it is important to validate the method and test its capabilities with healthy users.

Kinematic differences between the dominant and non-dominant hands are well documented in the literature (Batmaz et al. 2020; Sachlikidis and Salter 2007; Southard 2006; Sainburg 2002; Carson et al. 1997), with dominant arm performance generally being characterised by more efficient and less variable movements (Bagesteiro et al. 2020; Xiao et al. 2019; Schaffer and Sainburg 2017; Bagesteiro and Sainburg 2002). Bimanual circle drawing tasks are particularly effective in highlighting these differences, where non-dominant hands produce circles which are less round and more variable in size and shape (Nouredanesh et al. 2019; Carson et al. 1997). In addition to shedding light on manual asymmetries and neural control processes, comparisons between the two limbs are often included in the context of clinical research. Specifically, the differences between the dominant and non-dominant arms are used as benchmarks for comparison in clinical studies. The dominant hands of healthy participants are typically used as a comparison against the performance of the unaffected arm, whereas the non-dominant arm serves as a substitution for impairment in healthy controls, often being directly compared with the affected arm in patient populations (for example, see Vittersø et al. 2021; Johansson and Häger 2019; Lodha et al. 2013; Mansfield et al. 2011). As such, hand dominance may form an acceptable substitution for impairment where the recruitment of vulnerable or clinical groups is not feasible.

The study described in this paper compares the hand kinematics and performance of healthy participants using their dominant and non-dominant hands on a simple unimanual circle drawing task administered remotely through a virtual environment. VR-based assessments of upper limb function are an emerging field of research. However, to date there are few such tools which are immersive, portable, quick to administer, and low-cost (for examples, see Bank et al. 2018; Cidota et al. 2017; Gagnon et al. 2014), and we know of only one recent study which fits these criteria (Everard et al. 2022). A key highlight of our paper is the ability to collect motion capture data remotely and to investigate differences between the dominant and non-dominant hands without the need for a researcher to be present. With this study, we aim to demonstrate that a VR-based circle drawing task may be an effective and low-cost method to detect subtle differences in movement performance and function between the upper limbs, highlighting the potential role of remotely administered VR-based assessment techniques in healthcare.

Although the bulk of the prior research in circle drawing examines bimanual tasks, we chose to focus on unimanual circle drawing to better mirror the unimanual nature of current arm function tests (e.g. Box and Block Test: Mathiowetz et al. 1985; FMA: Fugl-Meyer et al. 1975) and to avoid issues related to attentional division (for example, attentional bias directed towards the dominant hand in bimanual tasks, see Buckingham and Carey 2009, 2015). Using the standalone Meta Quest VR HMD, the method is also portable, quick to administer, and not reliant on expensive computing hardware. The rationale, hypotheses, sampling plan, experimental method, and analysis plan for this study were preregistered on the Open Science Framework (OSF) (https://doi.org/10.17605/osf.io/t34uq) prior to beginning data collection (see Online Resource SM2 for a complete list of Transparent Changes). Our goal for this study was to investigate whether our method could detect subtle differences in movement performance between the two hands; and to discuss whether this may be a useful application for assessment in clinical populations. We hypothesised that circle drawing performance would differ between the two hands. Specifically, we expected that circles drawn with the dominant hand would be drawn faster, smoother, with a shorter hand-path; and that those circles would also be rounder than those drawn with the non-dominant hand. In addition, we also expected that circles drawn with the dominant hand would have less variation in size than those drawn with the non-dominant hand.

## Method

### Participants

47 participants (6 Female; $M_{{{\text{age}}}}$ = 35.6 years; *SD* = 10.8, *Range* = 18–54 years) who either owned or had access to a Meta Quest (Meta Platforms, Inc., California, USA) VR HMD were recruited from around the world through social media, email and word of mouth. Handedness was determined through a virtual version of the Edinburgh Handedness Inventory (EHI) short version (Veale 2014). If an individual was classed as ambidextrous on the EHI (i.e. a laterality quotient between − 60 and 60), their writing hand was used to determine their handedness. For example, participants classed as ambidextrous who specified that they mainly write with their right hand were classified as right-handed. One participant who scored as ambidextrous specified that they use both hands to write with and were subsequently removed from the analysis. Seven participants had incomplete datasets, containing only the consent form and/or a small number of trials, and were thus excluded from the analysis. Two further participants were removed following data cleaning (explained further below), leaving a final sample of 37 participants, four of whom were left-handed.

### Remote data collection and virtual environment

**Fig. 1:**
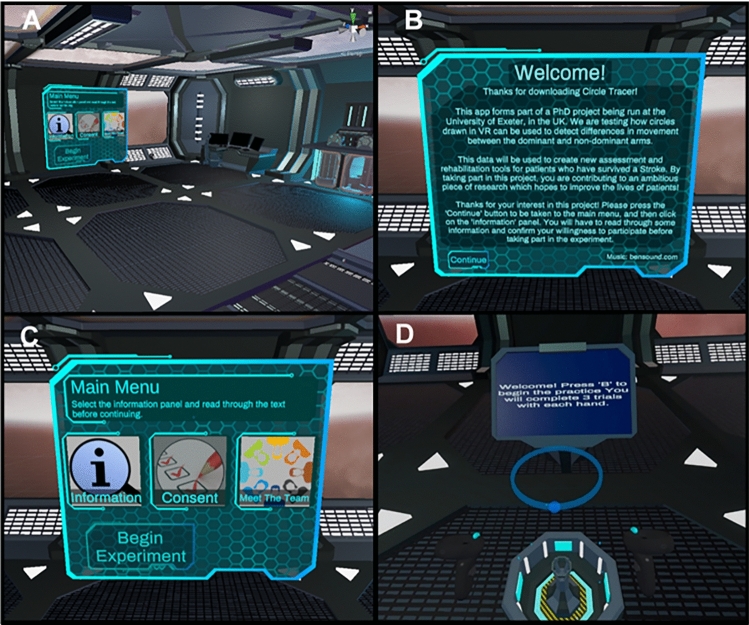
Images of the virtual environment, Circle Tracer. **A** The general environment, a spaceship-themed room complete with props and the Main Menu. **B** The screen shown to participants upon starting the app. **C** The Main Menu used to navigate the app, including buttons to access the information sheet, the consent form, information about the research team, and to begin the experiment. **D** The experimental task. The monitor is providing on-screen instructions, whilst the target circle is in the centre above a projector. The starting position is the sphere at the base of the circle, whilst the tips of the controller are indicated by the cyan spheres

In order to collect data throughout the COVID-19 pandemic, all aspects of this study—including consent, demographics and anonymised questionnaire responses—were administered entirely remotely through a custom-made VR environment: ‘Circle Tracer’. The environment was developed using the Unity game engine (Unity Technologies, San Francisco, USA) and was made available for participants to download onto their own devices using SideQuest (SideQuestVR, Belfast, UK). The environment consisted of a spaceship-themed room containing various props (Fig. 1). A holographic screen and menu were used to navigate the app. For the main task, participants were asked to trace the outline of a holographic circle oriented in the horizontal plane. Circle drawing in this plane has previously been used to characterise recovery from upper limb motor disorders (see Alves et al. 2022; Krebs et al. 2012; Krabben et al. 2011; and Dipietro et al. 2007), and it has also been shown to correlate well with the range of movement outwards from the body (Krabben et al. 2011). The holographic circle was created in Blender (Blender Foundation, Amsterdam, Netherlands) before being imported into Unity and had a 14 cm radius with a visible edge of approximately 1 cm^2^. Unity’s default scale is set so that one ‘unit’ of distance is equal to one metre in the real world, meaning that the radius of the holographic circle represents a real-world radius of 14 cm. A small transparent sphere at the front of the circle indicated where the participant should start and end their movements. The position of the circle was adjusted for each participant based on their arm length, with the centre of the circle appearing approximately 16 cm closer to the participant than their maximum forward reach. This ensured that the circle would always be within a comfortable reach of the participant. The height of the circle was approximately 1.4 m from the floor. A monitor was placed in front of the participant to present instructions on how to complete the task. Hand movements were tracked at approximately 72 Hz with the Meta Quest’s Insight tracking system, although this rate varied for each participant (see Sect. 2.5 for detail). Positional data were recorded using a custom C# script implemented in Unity. This script tracked the XYZ positions and rotations of the controllers held in each hand and periodically uploaded the data to a private server throughout the experiment using Unity’s UnityWebRequest method.

### Procedure

#### Informed consent

Participants were asked to read through an information sheet hosted online before downloading the Circle Tracer app from SideQuest. Upon starting the app, participants were presented with a ‘Welcome’ screen and a menu containing a virtual information sheet, information about the research team, and a consent form in the virtual environment. Participants were asked to read the information sheet again before ticking a series of boxes to provide informed consent using the built-in form. Participants were only able to access the experiment if they had completed the consent form.

#### Experiment

**Fig. 2:**
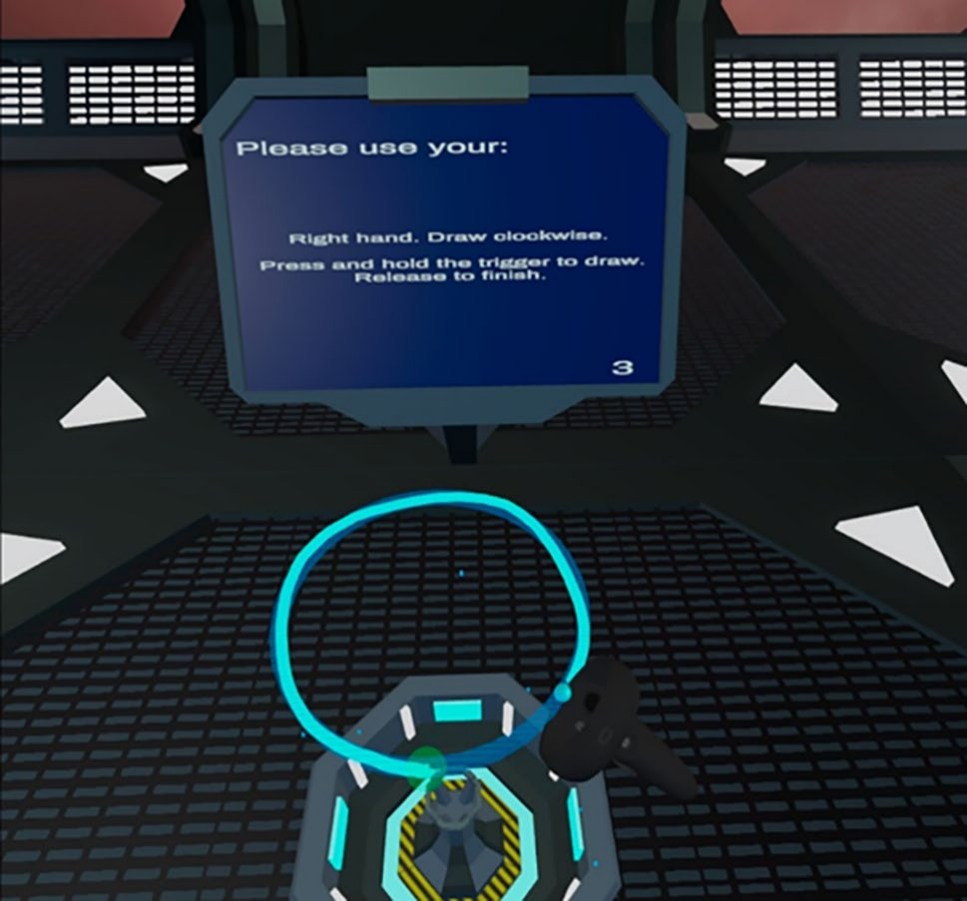
Example of a right-handed trial. The cyan line is the visual representation of the participant's hand path, displayed to participants in real time as they draw. The tip of the controller is indicated by the cyan sphere. A downloadable version of the experiment, along with a video showing an example of a trial, can be found on the Open Science Framework, at https://osf.io/zn3my/

After progressing to the experiment, a screen on the participant’s display provided an explanation of the task and some instructions on how to complete each trial. In order to avoid collisions with furniture, and to prevent participants resting on surfaces during trials, participants were asked to remain standing for the duration of the experiment. Participants were then asked to input their age and gender and to complete a virtual version of the EHI. This was administered using a Likert-scale slider to indicate their preference in the use of hands for various tasks. A six-second-long video was then presented, illustrating how to complete the task. Prior to beginning the experimental trials, participants completed six practice trials (three with the left hand and three with the right hand). To complete a trial, a participant had to place the tip of their controller in the start zone, hold down the trigger and complete one full revolution of the circle, until they returned to the starting position (Fig. 2). Data were not recorded from these practice trials. Participants then proceeded to the main experiment. This consisted of 32 trials: 16 each for the dominant and non-dominant hands. This number of trials was chosen through pilot testing to balance the number of measurements without being overly time-consuming or fatiguing. Hand use was randomised to avoid practice effects and the monitor in the environment provided instructions on which hand should be used. Despite being a unimanual task, participants were asked to move counter-clockwise and clockwise when using their left and right hands, respectively, in order to avoid any effects arising from asymmetry between the movements (for example, see Carson et al. 1997). Movement was recorded as soon as the participant pressed the trigger of their controller down and ended once the trigger was released. Trial data were uploaded to the server once each trial was completed.

Participants were instructed to “trace the outline of the circle as accurately and as quickly as you can”, with no emphasis placed on aspect one over the other. Visual and audio feedback were given to the participant while they were tracing the circle. A continuous humming noise was played, and a cyan-coloured line was drawn at the position of the participant’s hand while they held down the trigger. Upon release, the line disappeared, and the humming noise was replaced by a single musical tone to indicate the end of the trial. Participants were asked to repeat a trial if they strayed more than 7 cm away from the edge of the target circle (in any direction) at any time during a trial.

Finally, after finishing the main task participants were asked to complete a virtual version of the I-Group Presence Questionnaire (IPQ) (Schubert et al. 2001; Schubert 2003), a scale designed to assess presence or the sense of ‘being’ within a virtual environment. It is scored on a 7-point Likert scale and contains subscales for four components of presence: Spatial Presence (SP)—the feeling of being physically inside a space; Involvement (INV)—assessing engagement in the environment and awareness of the external world; Realism (REAL)—a notion of how realistic the environment and its components are; as well as a ‘General’ component measuring the broad feeling of ‘being there’. This measure was included as a form of feedback for the virtual environment, so that subsequent iterations of the task and environment can be improved. Results from this questionnaire can be viewed on the OSF page for this article (https://osf.io/j8xrt), and also in Online Resource SM3. In total, the experiment took approximately 15 min to complete.

### Data analysis

#### Kinematic data

Positional data taken from the controllers were filtered using a 2nd order, dual-pass, zero phase shift Butterworth filter with a 10 Hz cut-off (Franks et al. 1990). Data were then resampled at 90 Hz to ensure a uniform sampling rate between trials. Mean velocity on each trial, smoothness (defined as mean absolute jerk), movement time (time taken to complete a trial), and path length (cumulative distance in XYZ-space) were averaged for each participant. Prior to averaging, 1% of the total frames recorded on each trial were removed from the beginning and end to avoid artefacts arising from differentiation. For instances where participants had pressed the trigger down but had delayed starting their movement, we identified movement onset by detecting when velocity in the x-axis first exceeded 50 mm per second for three consecutive frames (Arthur et al. 2021; Eastough and Edwards 2007). For exploratory analysis of the hand kinematics (see Online Resource SM1 for figures), peak velocity, peak and mean acceleration, path length in the Y-axis as well as mean and variance of velocity in the Y-axis were averaged for each participant.

#### Circle metrics

**Fig. 3:**
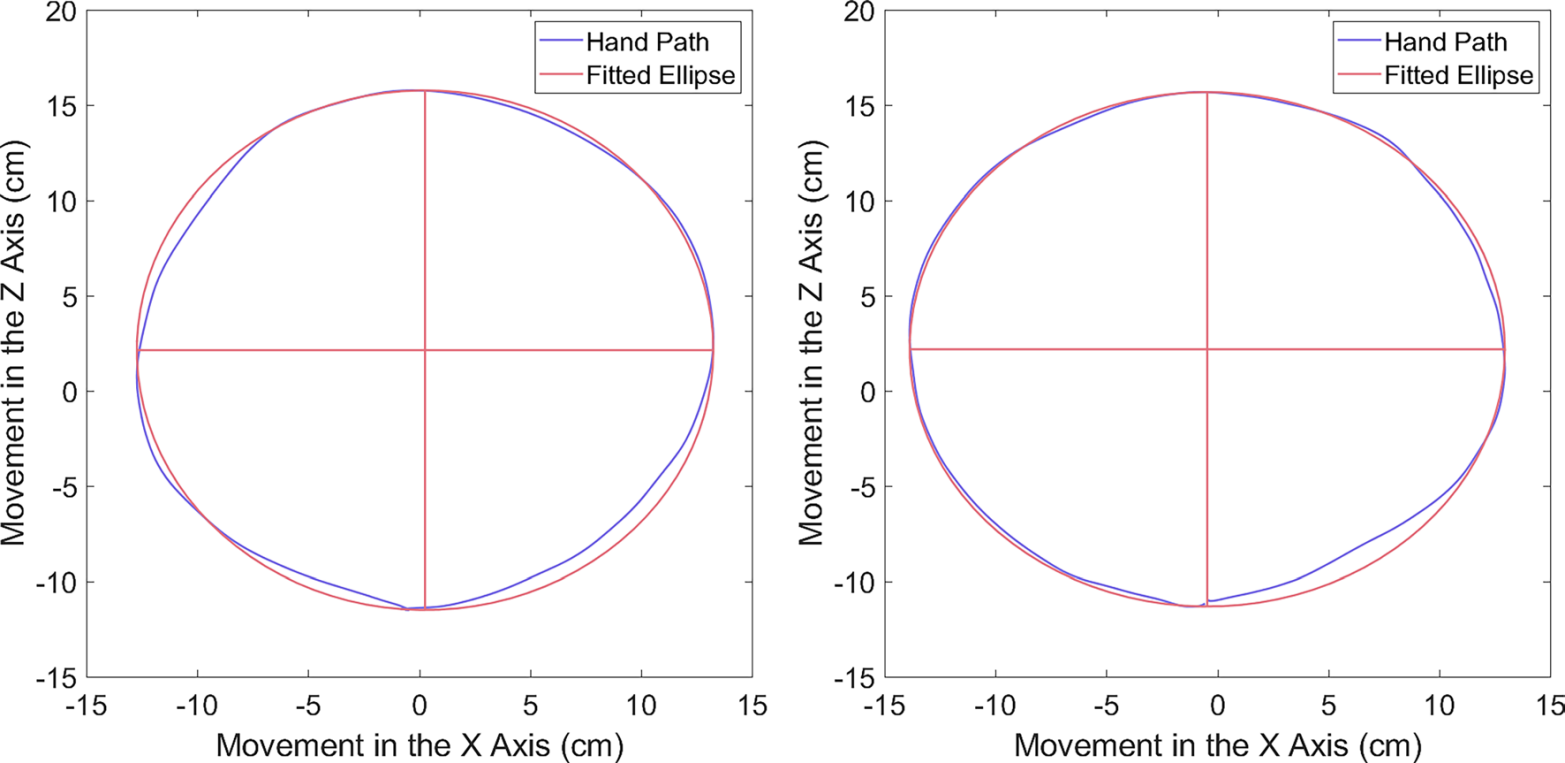
Examples of hand drawn circles and the corresponding ellipses, fitted through PCA. Examples are from two separate trials of a single participant

To calculate our circle metrics, we fitted a mathematically generated ellipse to the participant’s hand path. For our main analysis, we used Principal Component Analysis (PCA) to generate and fit the ellipses (see Tuță et al. 2019 for method). Size-variance and roundness of the drawn circles were averaged for each participant. Size-variance was calculated as the standard deviation in circle area across each participant’s trials. Area is calculated as:

$$A = \pi ab$$

where *a* and *b* are the major and minor axes of the fitted ellipse, respectively. Circle roundness was calculated as the ratio between the minor and major axes of the fitted ellipse for each participant (as in Krabben et al. 2011; Oliveira et al. 1996). This ratio has a value between zero and one, with values closer to one representing a more perfect circle. Examples of some fitted ellipses are shown in Fig. 3.

### Data treatment and analysis

**Fig. 4:**
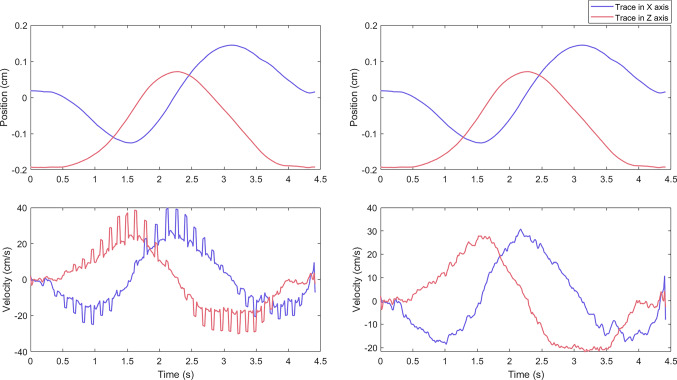
Positions (Top) and velocities (Bottom) in the X (purple) and Z (red) axes from a trial containing artefacts due to missing frames. The figures on the left of the panel, depict data prior to cleaning. The figures on the right depict data which has been cleaned but not yet filtered

Position and velocity traces for each trial were visually examined by one of the authors to ensure data quality. One participant exhibited high levels of noise on all trials both before and after applying the chosen Butterworth filter, evidenced by the presence of rapid peaks and troughs uncharacteristic of smooth movement. As a more stringent filtering procedure (e.g. reducing the cut-off) may have affected interesting features in the movement signal of all trials in our sample, rather than just those of the noise, we instead opted to remove the affected trials from the analysis. In several of our datasets a number of trials contained artefacts resembling regular, sudden step-like jumps in position and velocity. Trials affected by these artefacts recorded a framerate lower than that of the 72 Hz typical with the Meta Quest. These artefacts were assumed to be missing frames possibly caused by poor lighting conditions, where too much natural or infrared light can cause interference and prevent effective tracking of the controllers (see Melim 2022, for example). As the artefacts spanned only single frames, a simple linear interpolation technique was used to impute missing points. Due to the recording frequency of the Meta Quest, under normal circumstances, the time difference between each frame should be consistent (around 0.0138 s for a 72 Hz recording rate). However, if frames are missing, then this value should temporarily increase until the regular recording-rate resumes. Therefore, missing frames were first detected by scanning position and velocity traces for sudden increases in the time-step which were more than 1.5 times the mean time-step value. These missing frames were then filled by interpolating between the previous frame and the subsequent frame (Fig. 4). In order to preserve data accuracy, trials which contained enough missing frames to result in an overall framerate below 60 Hz were excluded from the analysis. This avoided imputing data on trials where little information was available, thereby potentially creating inaccurate results. Furthermore, trials were inspected quantitatively and qualitatively to ensure they had been completed correctly. Quantitatively, if trials had been completed in less than 300 ms, had a roundness value of less than 0.3, or had a path length less than 50 cm, they were deemed to have been completed early and were removed from the sample. In addition, the three-dimensional (xyz) positional values of the participants hand path were plotted and visually inspected for clear indications of improper behaviour (e.g. drawings that were not circles: lines, squares, scribbles, etc.). As in our preregistration, data that could be considered outliers (i.e. appearing over 3.5 standard deviations above/below the median or mean) were not excluded unless they fulfilled the criteria outlined above. Overall, 66 trials were rejected (64 of which were from two separate participants), resulting in a final sample of 37 participants and a total of 592 trials for the dominant hand and 590 trials for the non-dominant hand being used in the final analysis.

Data analysis was performed in MATLAB (version R2019b, The MathWorks, Inc., Massachusetts, US). Data were checked for normality using the Shapiro–Wilk Normality Test. Performance between the dominant and non-dominant hands was compared through a series of paired samples *t* tests and Wilcoxon signed-rank tests where normality was violated. Effect sizes for *t* tests were calculated as Cohen’s *d*, whilst those for Wilcoxon signed-rank tests, *r*, were calculated by dividing the *Z*-value by the square root of the number of samples (Tomczak and Tomczak 2014; Fritz et al. 2012).

## Results

### Main analysis

Our main hypotheses were that circle drawing performance would differ between the two hands, such that the dominant hand would draw circles faster, smoother, with a shorter hand path; and that those circles would be more circular. We also hypothesised that the dominant hand would produce circles less variable in size, as indicated by a smaller average Standard Deviation in circle area. Shapiro–Wilks test for normality was violated on all metrics except path length. As such, Wilcoxon Signed-Ranks tests were used as the non-parametric alternative. Accordingly, we report the median and the interquartile range for each of these tests, rather than the mean and standard deviation. Due to the presence of multiple comparisons, Bonferroni corrections were applied to all tests in the main analysis to minimise type 1 error. We include six tests in these main analyses and set the altered alpha level, *a*, to 0.0083.

**Fig. 5:**
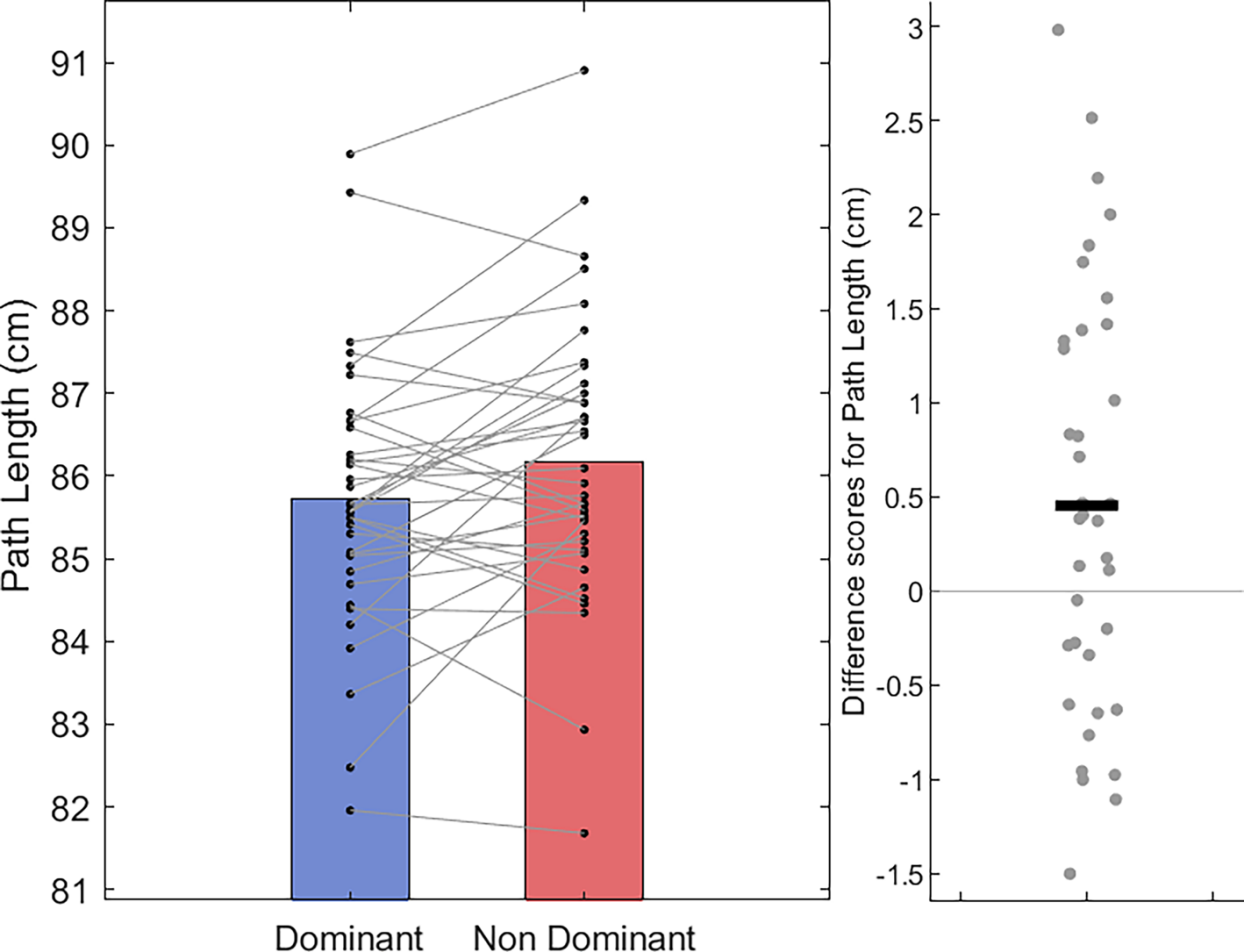
Bar and scatter plots with individual matched data points, showing the difference in path length for the dominant hand compared to the non-dominant hand. Panel B shows the difference scores with the black bar representing the mean difference

**Fig. 6:**
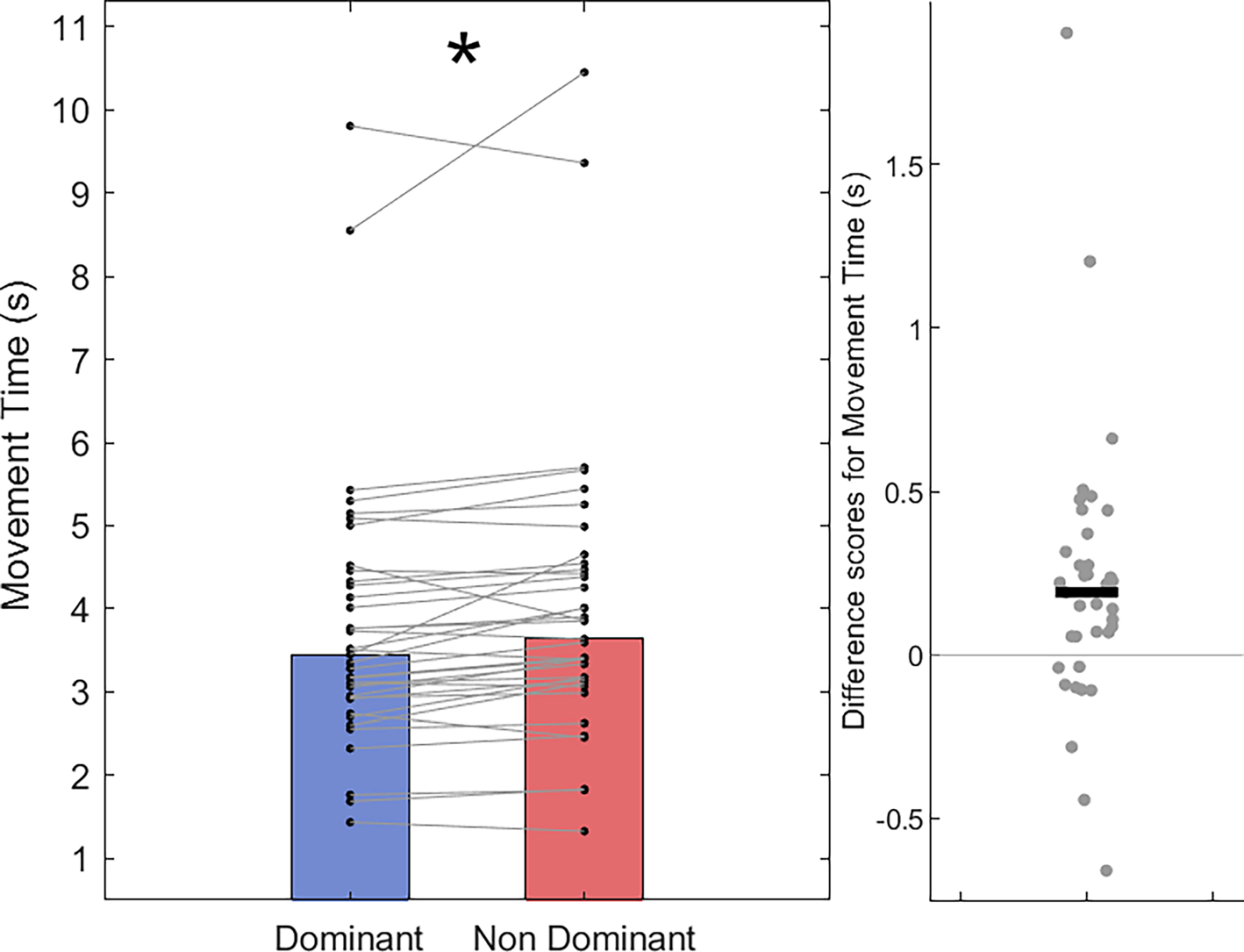
Bar and scatter plots with individual matched data points, showing the difference in movement time for the dominant hand compared to the non-dominant hand. Panel B shows the difference scores with the black bar representing the median difference. Asterisk shows significance

**Fig. 7:**
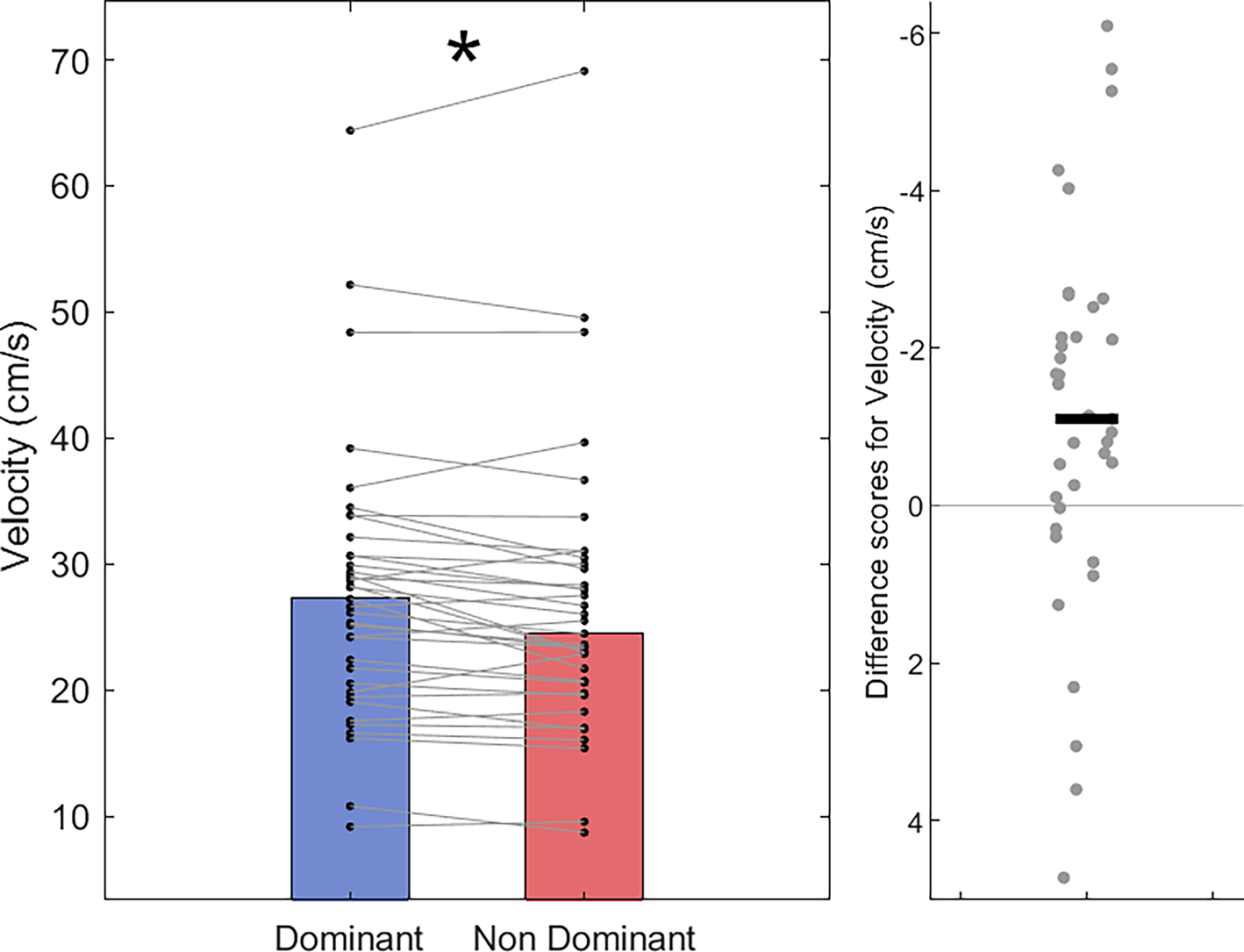
Bar and scatter plots with individual matched data points, showing the difference in mean velocity for the dominant hand compared to the non-dominant hand. Panel B shows the difference scores on a flipped axis, with the black bar representing the median difference. Asterisk shows significance

**Fig. 8:**
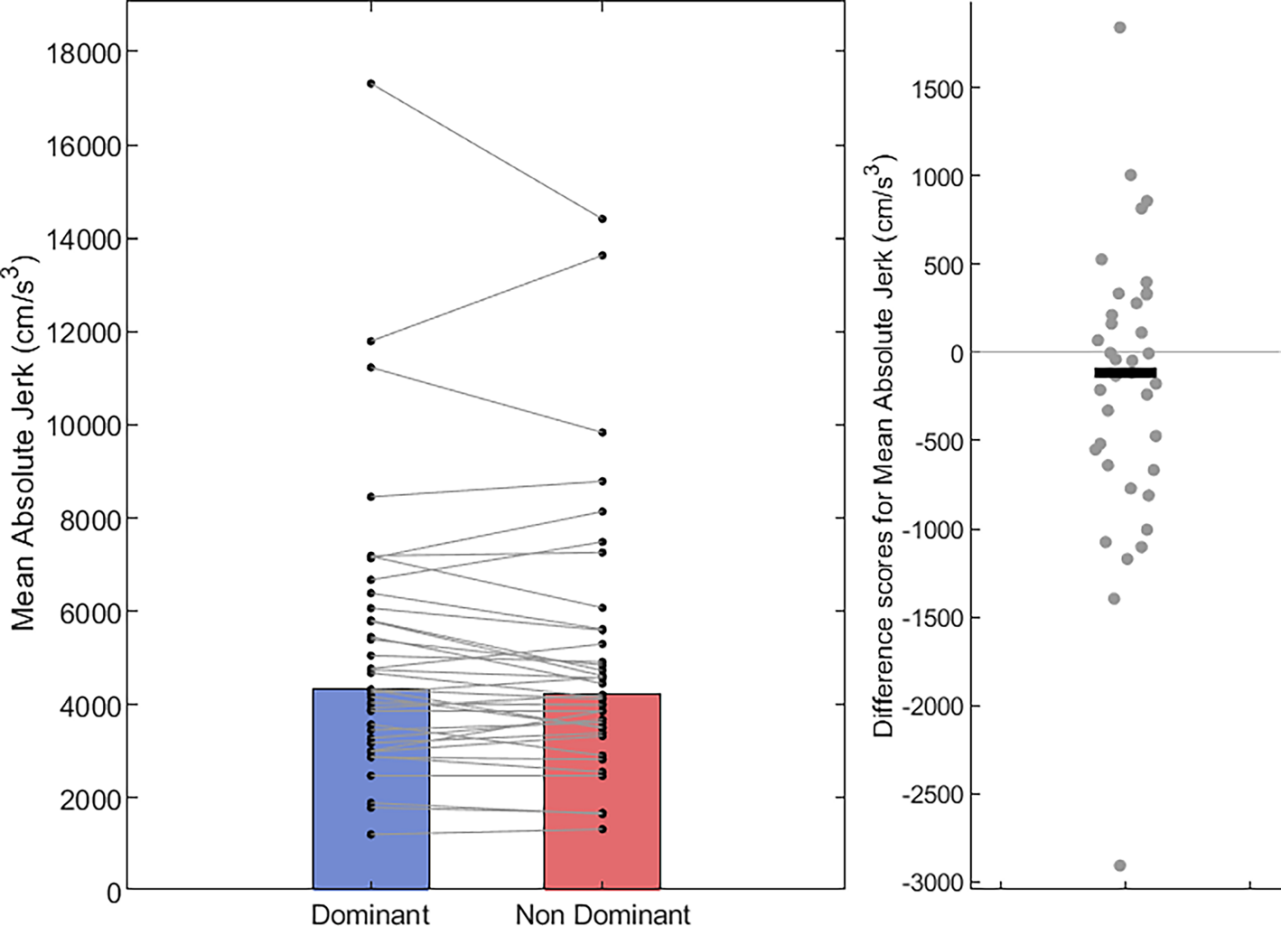
Bar and scatter plots with individual matched data points, showing the difference in mean absolute jerk (movement smoothness) for the dominant hand compared to the non-dominant hand. Panel B shows the difference scores with the black bar representing the median difference

After correcting for multiple comparisons, a paired-sample *t* test showed no difference in path-length between the dominant hand (*M* = 85.72 cm; *SD* = 1.58 cm) compared with the non-dominant hand (*M* = 86.17 cm; *SD* = 1.72 cm), *t*(36) = − 2.47, *p* = 0.018, *d* = − 0.41 (Fig. 5). In terms of movement time, Wilcoxon Signed-Rank tests show that dominant hand trials (Mdn = 3.45 s, IQR = 1.43 s) were completed in less time than non-dominant hand trials (Mdn = 3.64 s, IQR = 1.36 s); *T* = 132.5, *z* = − 3.30, *p* < 0.001, *r* = − 0.53 (Fig. 6). Accordingly, mean velocity was higher on trials using the dominant hand (Mdn = 27.23 cm/s, IQR = 10.70 cm/s) than those using the non-dominant hand (Mdn = 24.47 cm/s, IQR = 9.74 cm/s); *T* = 536, *z* = 2.78, *p* = 0.005, *r* = 0.45 (Fig. 7). For movement smoothness (mean absolute jerk), results show no evidence of a difference between the two hands (Dom Mdn = 4323 cm/s^3^, IQR = 2885 cm/s^3^ vs Non-dom Mdn = 4206 cm/s^3^, IQR = 2122 cm/s^3^; *T* = 444, *z* = 1.40, *p* = 0.16, *r* = 0.22, Fig. 8).

**Fig. 9:**
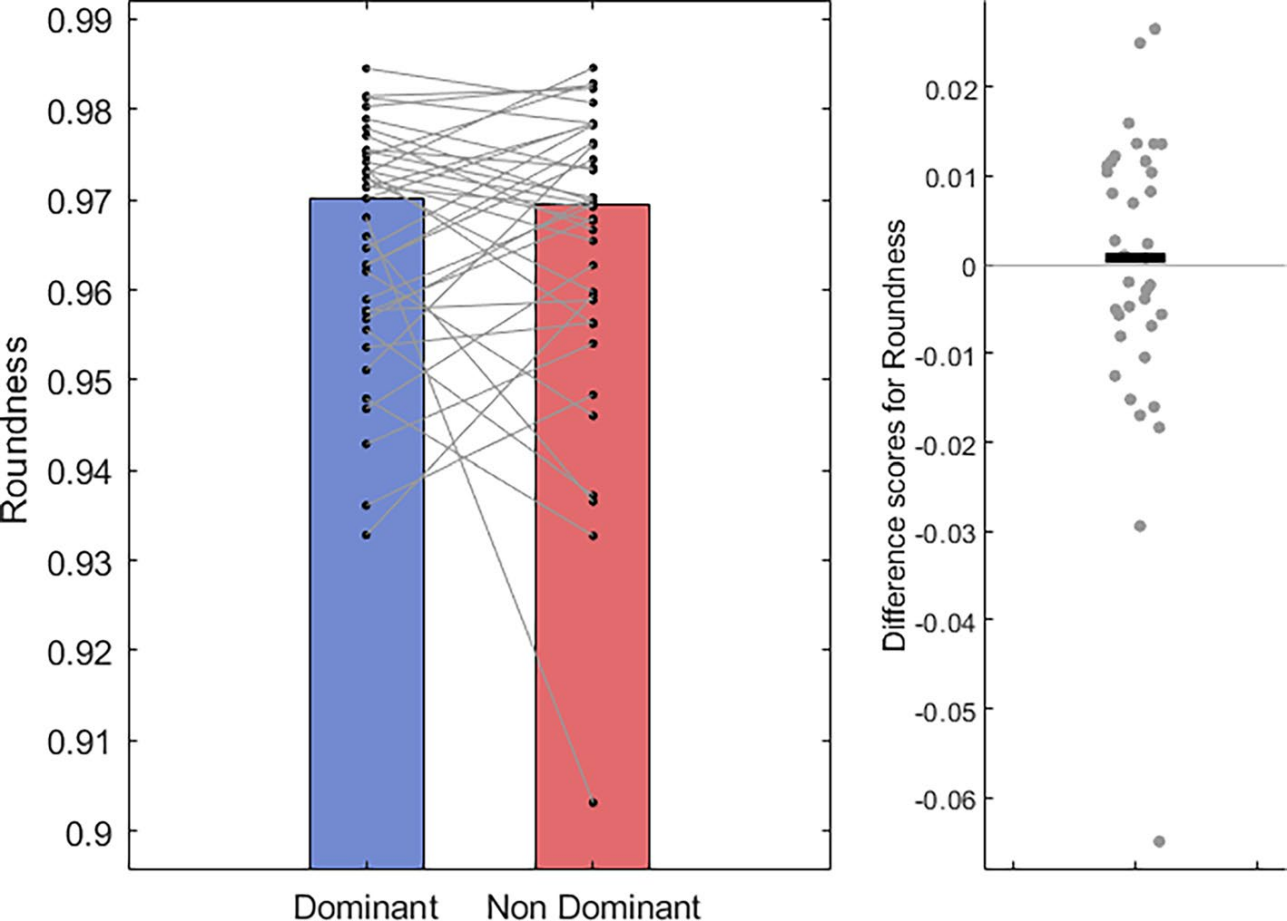
Bar and scatter plots with individual matched data points, showing the difference in mean roundness (from 0 to 1) of ellipses fitted to the participants hand path for the dominant hand compared to the non-dominant hand. Panel B shows the difference scores with the black bar representing the median difference

**Fig. 10:**
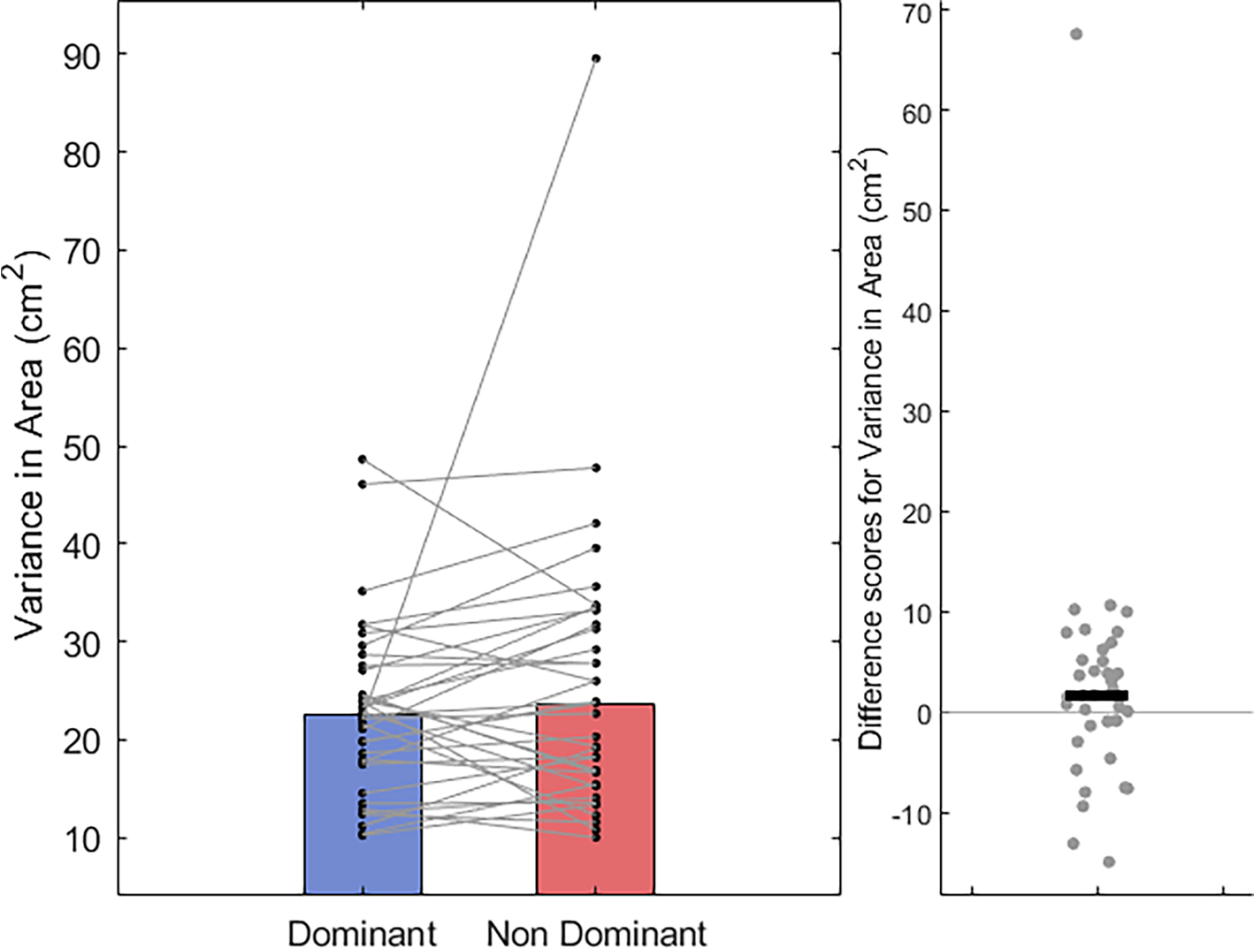
Bar and scatter plots with individual matched data points, showing the difference in area variance, measured as the mean standard deviation in circle area between the dominant hand compared to the non-dominant hand. Panel B shows the difference scores with the black bar representing the median difference

Moreover, there was no evidence of a difference in circle roundness (Dom Mdn = 0.97, *IQR* = 0.02 vs Non-dom Mdn = 0.97, *IQR* = 0.02; *T* = 334, *z* = − 0.26, *p* = 0.79, *r* = − 0.04, Fig. 9); nor in the variation of circle area (Dom Mdn = 22.45 cm^2^, IQR = 9.50 cm^2^ vs Non-dom Mdn = 23.70 cm^2^, IQR = 15.78 cm^2^; *T* = 254, *z* = − 1.47, *p* = 0.14, *r* = − 0.24, Fig. 10) between the two groups. It may be important to note the inclusion of potential outliers in the analysis of these two metrics. An additional analysis with these data points removed can be found in the supplementary materials (Sects. 1.2, Online Resource SM1). Overall, although we find little evidence for our main hypotheses that unimanual circle-drawing performance differs between the dominant and non-dominant hands, we do find evidence that hand kinematics differ; where circles are drawn at a faster rate with the dominant hand.

### Exploratory analysis

After our main analyses, we also explored other potential kinematic differences between the two groups. As these metrics are exploratory and were not driven by specific pre-planned hypotheses, alpha-level corrections were not applied to these results (as advised in Armstrong 2014). Aside from Path Length in the Y-axis, Shapiro–Wilks test for normality was violated on all measures and, accordingly, Wilcoxon-Signed Ranks tests were used as the non-parametric alternative.

Although circles were drawn in the horizontal plane, these movements were unsupported against gravity. Therefore, we were also interested in whether hand-use would affect movement stability (i.e. maintenance of position and speed) across the vertical plane, indicated by variations in speed and position in the Y-axis. We first tested differences of path length in the Y-axis between the dominant and non-dominant hands (Supplementary Fig. 1, Online Resource SM1). A paired samples *t* test indicated that path length in the Y-axis was shorter for the dominant hand (*M* = 6.76 cm, *SD* = 1.21 cm) than for the non-dominant hand (*M* = 7.36 cm, *SD* = 1.25 cm), *t(36)* =  − 4.40, *p* < 0.001, *r* = − 0.72. Next, we checked mean velocity and the variance in velocity on the Y-axis between the two groups (Supplementary Figs. 2 and 3, Online Resource SM1). Velocity in the Y-axis did not significantly differ between the two hands (Dom Mdn = 1.97 cm/s, IQR = 0.84 cm/s, vs Non-dom Mdn = 1.99 cm/s, IQR = 0.98 cm/s), *T* = 257, *z* = − 1.43, *p* = 0.15, *r* = − 0.23. Additionally, dominant and non-dominant hands were equally variable in speed (Dom Mdn = 1.60 cm/s, IQR = 0.69 cm/s, vs Non-dom Mdn 1.53 cm/s, IQR = 0.65 cm/s), *T* = 333, *z* = − 0.28, *p* = 0.78, *r* = − 0.05.

We also checked for differences in the peak velocity (Supplementary Fig. 4, Online Resource SM1) and the mean and peak acceleration between the two groups. Unlike mean velocity, trials completed with the dominant hand (Mdn = 40.20 cm/s, IQR = 17.90 cm/s) did not have a significantly higher peak velocity than those with the non-dominant hand (Mdn = 37.28 cm/s, IQR = 14.57 cm/s), *T* = 470, *z* = 1.79, *p* = 0.07, *r* = 0.29. Similarly, there was no significant difference in mean acceleration between the two groups (Dom Mdn = 54.78 cm/s^2^, *IQR* = 32.79 cm/s^2^, vs Non-dom Mdn = 56.54 cm/s^2^, IQR = 26.90 cm/s^2^), *T* = 379, *z* = 0.42, *p* = 0.68, *r* = 0.07 (Supplementary Fig. 5, Online Resource SM1). Finally, results also indicate that peak acceleration was equal between dominant hand trials (Mdn = 452.54 cm/s^2^, IQR = 274.70 cm/s^2^) and non-dominant hand trials (Mdn = 398.46 cm/s^2^, IQR = 147.25 cm/s^2^), *T* = 469, *z* = 1.77, *p* = 0.08, *r* = 0.28 (Supplementary Fig. 6, Online Resource SM1).

## Discussion

There is a need for assessments of upper-limb function which are objective and detailed. For their implementation to be feasible, outcome measures need to be easy to set up and quick to administer. Current clinical assessments are subjective and limited by ceiling effects (see Lamers et al. 2013; Platz et al. 2005) where patients commonly reach the highest measurement score, whereas technology-based outcome measures are objective but expensive and difficult to implement for the majority of clinical settings (Shirota et al. 2019). This study examined a novel VR-based method of detecting functional differences in the upper limb, which is quick to complete, low cost, and portable.

We tested whether a circle drawing task, administered remotely to participants through the Meta Quest, would be able to detect differences between the dominant and non-dominant arms of healthy participants. We found some evidence to support our hypotheses that hand kinematics would differ between the dominant and non-dominant hands of healthy participants, and that these differences can be detected by a remotely administered VR-based circle drawing task. With regard to movement time and mean velocity, participants completed trials in less time with their dominant hand and moved faster on average with their dominant hand. For our measure of path length, we noted that the dominant hand travelled a shorter distance than non-dominant hands. It is worth noting, however, that after correcting for multiple comparisons this difference did not reach significance but may nonetheless represent an interesting direction for future investigation. We found no difference in movement smoothness (defined as the mean absolute jerk) between the two limbs. In addition, there was no apparent difference between the variation in size and roundness of the circles drawn with either hand.

In our VR circle drawing task, we observed differences between the dominant and non-dominant hand in both temporal (e.g. movement time and velocity), and spatial (e.g. path length, path length in the Y-axis), measures of circle drawing performance. However, we failed to find differences in key spatial measures such as size and roundedness, as well as in movement smoothness. These findings appear difficult to reconcile with previous works on circle drawing (Nouredanesh et al. 2019; Summers et al. 2008; Byblow et al. 1999; Carson et al. 1997), and however, one reason for this apparent discrepancy with past literature could be the different nature of our task. The majority of past work utilises bimanual tasks in which circles are drawn continuously with both hands, often while matching the pace of a metronome (e.g. Repp 2011; Tseng and Shulz 2005). The introduction of constraints such as timing and/or bimanual movement can have significant impacts on circle drawing performance, such as an increase in error and a reduction in the roundness of circles, particularly at higher speeds (Pfann et al. 2002; Byblow et al. 1999) and especially so in the non-dominant hand (Lewis and Byblow 2004; Byblow et al. 1999). By contrast, the task we present in the current manuscript is of a considerably different nature: a self-paced, unimanual task with few other constraints. Participants drew circles discontinuously, each separated by an untimed break. As such, it is likely that our task was simply not difficult enough to elicit significant differences between the two hands across roundness and size measures. This can be reinforced by the expanded minimum jerk model proposed by Wann et al. (1988). This model suggests that when movement constraints are relaxed in circle drawing tasks (as in our task: unimanual, self-paced movement), it can be expected that maximising movement smoothness becomes a priority. In tasks which have additional constraints (either temporal or spatial), performance may be driven instead by the need to meet these constraints — for example, sacrificing accuracy and smoothness in order to preserve timing. In our case, it would appear that our participants are making efforts maximise the smoothness and roundness of their movements and that, particularly in the case of the non-dominant hand, this may be at the cost of speed (as evidenced by lower speeds in the non-dominant hand).

Furthermore, the outcomes we report are consistent with studies conducted within a similar, unimanual context. The dominant hand presents a shorter movement time and/or a higher velocity on a range of discontinuous unimanual tasks, such as reaching (Bagesteiro et al. 2020; Mieschke et al. 2001; Elliot et al. 1993), line drawing (Vuillermot et al. 2009), throwing (Sachlikidis and Salter 2007; Southard 2006) and those of manual dexterity (Temporiti et al. 2022; Bryden et al. 2007; Perderson et al. 2003; Annett et al. 1979); particularly so when there is an emphasis on accuracy rather speed.

Aside from the main analysis, we also conducted an exploratory analysis to compare the movement stability in the vertical axis (orthogonal to the plane of movement) between the dominant and non-dominant hands. Here, we observed that movements made with the dominant hand travelled less distance in the vertical axis, indicating that movements unsupported against gravity appear to be more stable when performed with the dominant hand. Interestingly, however, we found no differences between the speed and variability in speed of movements in the vertical plane. Furthermore, we also found that overall peak velocity, as well as peak and mean acceleration, was equal between the movements of the two hands.

It is important to note that, despite their significance, the differences observed in this paper are rather small (e.g. path length in the Y-axis showed a median difference of 0.6 cm). Dominant vs. non-dominant hand differences are well described across a large body of research (Schaffer and Sainburg 2017; Bagesteiro and Sainburg 2002; Sainburg 2002), and although our results may add to this body of work, the purpose of this experiment was not to discover novel indices of hand-dominance or manual laterality. Instead, this paper aimed to investigate whether a remotely administered VR-based circle drawing task can detect subtle differences in movement performance in the upper limb; and to consider whether this concept can be applied as a clinical assessment tool. As such, it can be argued that the small differences observed in this paper are precisely why this method has value. Many currently used assessments are less sensitive to change and can struggle to identify small but important changes in function following rehabilitation interventions (Alt Murphy et al. 2015; Catz et al. 1997). By comparison, we demonstrate a method which is capable of detecting subtle performance differences between the arms of healthy individuals, in both the spatial and temporal domains. At the time of writing, this method is comparable in cost to several clinical outcome measures such as the Box and Block Test (Professional “Box and Block” Test Kit, n.d.) and the Action Research Arm Test (Action Research Arm Test Kit, n.d.). Indeed, based on these results, we foresee a range of specific use cases for future iterations of our task, such as detecting small but important changes after interventions or attempting to screen individuals who have not yet been diagnosed with a particular disability or functional impairment. Given that circle drawing metrics have previously been shown to correlate with measures of stroke severity and upper limb function (Krabben et al. 2011), it is possible that this method can also be applied as a stratification tool: categorising patients into different functional groups based on their circle-drawing performance.

Although we have conducted this research in the context of assessment after stroke, it is possible that the method can also be applied to the quantification of movement performance in other clinical populations such as those with Cerebral Palsy or Developmental Coordination Disorder (DCD). A noteworthy characteristic of this method is the ability to assess differences in a portable and remote manner. This could be a particularly promising tool in the case of DCD, a condition which is often under-recognised in educational and care settings (Blank et al. 2019; Wilson et al. 2013). Given that DCD is often identified in the earlier years of primary school (Hunt et al. 2021), there could be value in schools having access to an on-premises screening tool which is low cost and portable and can be taken home by families to be used remotely.

There are some limitations to this paper which need to be addressed. First, participants were instructed to complete the task while standing. Although this may have avoided collisions and prevented participants resting on nearby surfaces, there is a question of whether participants would have been more stable if they remained seated. It is possible that our results may be influenced by fluctuations in postural sway—that is, small movements that are automatically made to maintain balance. However, the task we present was a self-paced stationary task conducted with participants who are presumably experienced in the use of VR headsets. Given that postural instability can improve with repeated VR exposure (Fransson et al. 2019), it is unlikely that the amount of postural sway experienced by participants in our sample would affect our results severely.

A second potential limitation is that due to the remote nature of the study, participants performed the task unsupervised. When the app was built, it was designed with various restrictions to ensure correct completion of the task. For instance, the app recognised only the left controller during a left-hand trial; and only the right controller during a right-hand trial. Furthermore, participants were asked to redo a trial if they had moved too far from the circle than was necessary. The data were thoroughly inspected to ensure complete correction of the task; and any trials which appeared to be incomplete or performed incorrectly were removed. Indeed, as participants were unsupervised, we ultimately do not know how they conducted the task beyond the simple end-effector kinematics that were recorded. However, as the circles we recorded in this experiment were almost perfect (having roundness values very close to 1), it can be assumed that participants generally completed the task using appropriate coordination of the shoulder and elbow joints. We see no reason to assume that participants failed to follow the task-instructions carefully and completed the task in the manner that was requested.

Nonetheless, there are several small changes that can be made to future iterations of the task, which may improve both the quality and usefulness of the data. First, it will be useful to collect continuous positional data of the participants head, derived from the position of HMD. This would provide a reference point for the height of the participant and could be used to quantify the extent of movement of the torso, in addition to the movement of the upper limb(s). Second, providing further instructions on how to complete the movement (e.g. “Try to move using only your upper limb, not by compensating with the torso) could potentially refine the specificity of the task, and the subsequent measurements that can be derived. As a last point, it may also be useful for participants to complete the task strictly while sitting; or to include both a sitting and a standing component so that the differences between these two factors can be compared. This would be particularly useful during the assessment of certain clinical populations and may allow for greater differentiation of function within groups (e.g. participants exhibiting better/worse upper limb movement performance while sitting/standing).

In conclusion, this paper has shown that VR circle drawing is quick to administer and can distinguish subtle differences between movement kinematics which traditional tests of clinical arm function might miss (Alt Murphy et al. 2015). This method is portable, can be conducted in person or remotely, and is of comparable cost with current observation-based assessments in an arguably less-subjective fashion. As such, it holds great potential for assessing upper-limb function in clinical populations with acquired or developmental movement difficulties. On the basis of these findings, we suggest that future research should utilise the benefits of portable VR HMDs to investigate movement differences in clinical populations who exhibit difficulties with upper-limb function and coordination. Specifically, researchers should attempt to validate a VR-based circle drawing task in a clinical population (e.g. stroke, CP or DCD), with comparisons to performance on current clinical assessments such as the FMA or the Box and Block Test. Furthermore, we identify a number of areas on which this task can be improved; and these should be considered in the future research to increase the applicability and quality of data derived from this method. In order to minimise the barriers faced in clinical settings (Shirota et al. 2019), it is of equal importance to assess the feasibility and usability of any technology-based assessment or intervention. As such, future VR-based assessments should be developed in line with patient and clinical needs to ensure that tasks are feasible to administer and that outcomes are relevant and useful to the end-users.

## Supplementary Information

Below is the link to the electronic supplementary material.

Supplementary file1 (DOCX 1033 KB)

Supplementary file2 (PDF 118 KB)

Supplementary file3 (DOCX 15 KB)

## Data Availability

The datasets containing the variables analysed during the current study are available in the Open Science Repository, at https://osf.io/zn3my/.
