# Supplementary material for: Using immersive virtual reality to remotely examine performance differences between dominant and non-dominant hands
Source: Virtual Real. 2023 May 6:1–16. Online ahead of print. doi: 10.1007/s10055-023-00794-z (PMC10162902; doi:10.1007/s10055-023-00794-z)
Supplement: Supplementary file 1 — Supplementary file1 (DOCX 1033 KB) [file 10055_2023_794_MOESM1_ESM.docx]

**Using Immersive Virtual Reality to remotely examine performance differences between dominant and non-dominant hands.**

***Virtual Reality***

Jack Owen Evans_­_­­^1^, Krasimira Tsaneva-Atanasova_­_^2,3^, Gavin Buckingham^1^

­^1^ Department of Sport and Health Sciences, Richards Building, Magdalen Road, University of Exeter, Exeter, Devon, United Kingdom, EX2 4TA

^2^ Department of Mathematics and Statistics, Living Systems Institute, University of Exeter, Exeter, Devon, United Kingdom, Exeter EX4 4QD
^3^ EPSRC Hub for Quantitative Modelling in Healthcare, University of Exeter, Exeter, Devon, United Kingdom, Exeter EX4 4QD

Jack Owen Evans – Corresponding Author

[je426@exeter.ac.uk](mailto:je426@exeter.ac.uk)

# Supplementary Materials

## Supplementary Figures for Exploratory Analyses


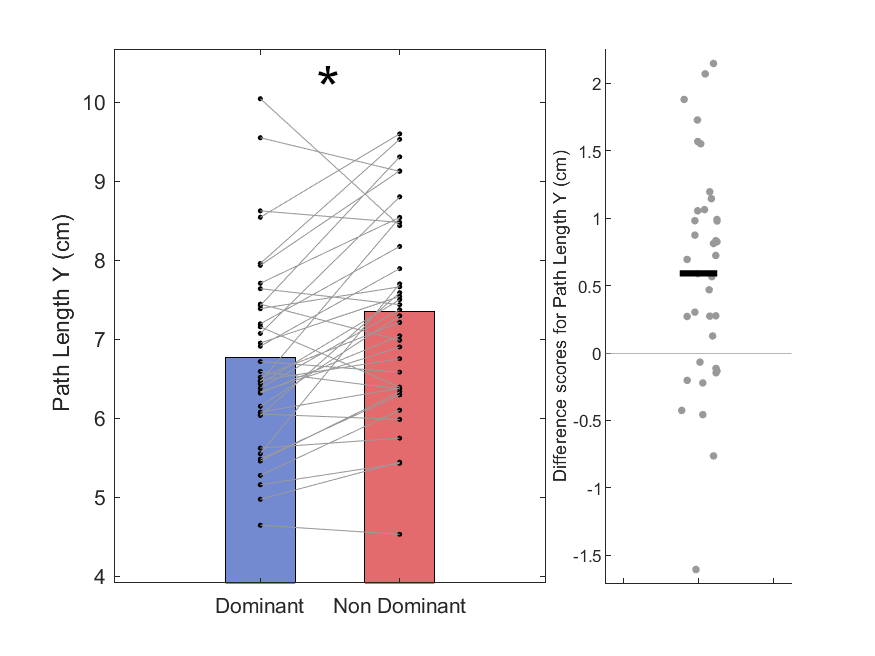


Supplementary Figure 1: Bar and scatter plots with individual matched data points, showing the difference in path length in the Y axis between the dominant hand compared to the non-dominant hand. Panel B shows the difference scores with the black bar representing the mean difference. The asterisk indicates significance.


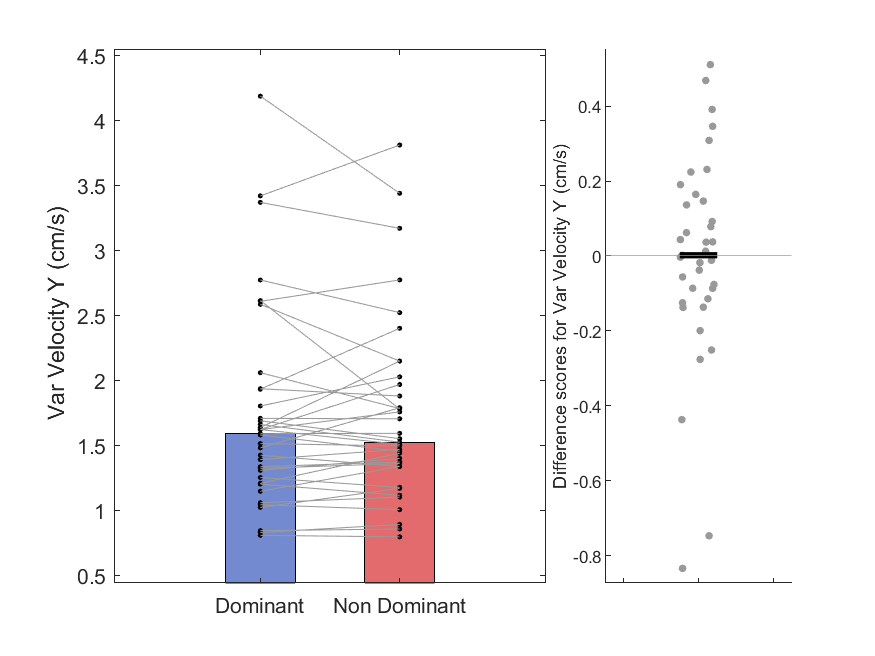


Supplementary Figure 3: Bar and scatter plots with individual matched data points, showing the difference in mean standard deviations for velocity (variance in speed) in the Y axis between the dominant hand compared to the non-dominant hand. Panel B shows the difference scores with the black bar representing the median difference.


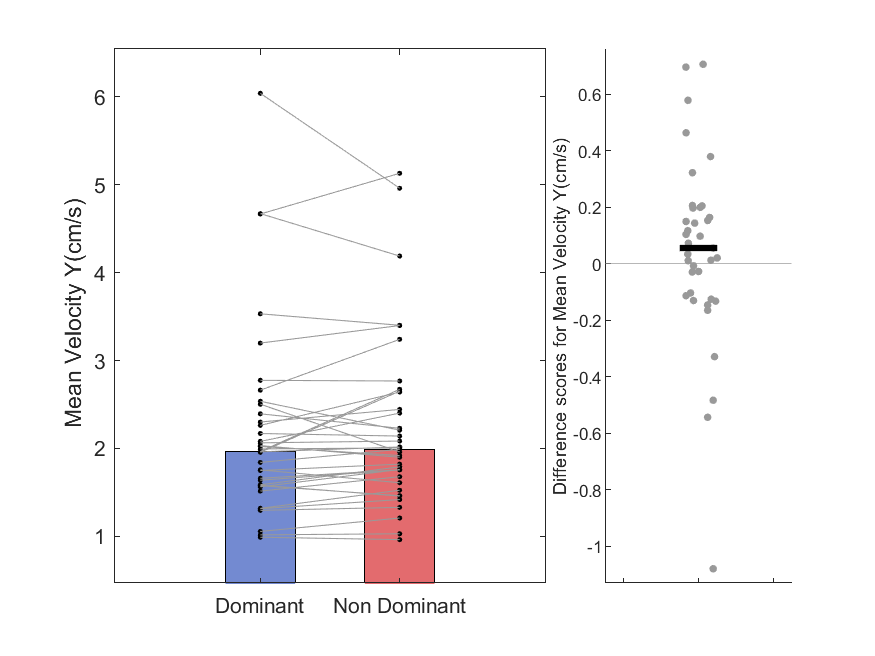


Supplementary Figure 2: Bar and scatter plots with individual matched data points, showing the difference in mean velocity in the Y axis between the dominant hand compared to the non-dominant hand. Panel B shows the difference scores with the black bar representing the median difference.


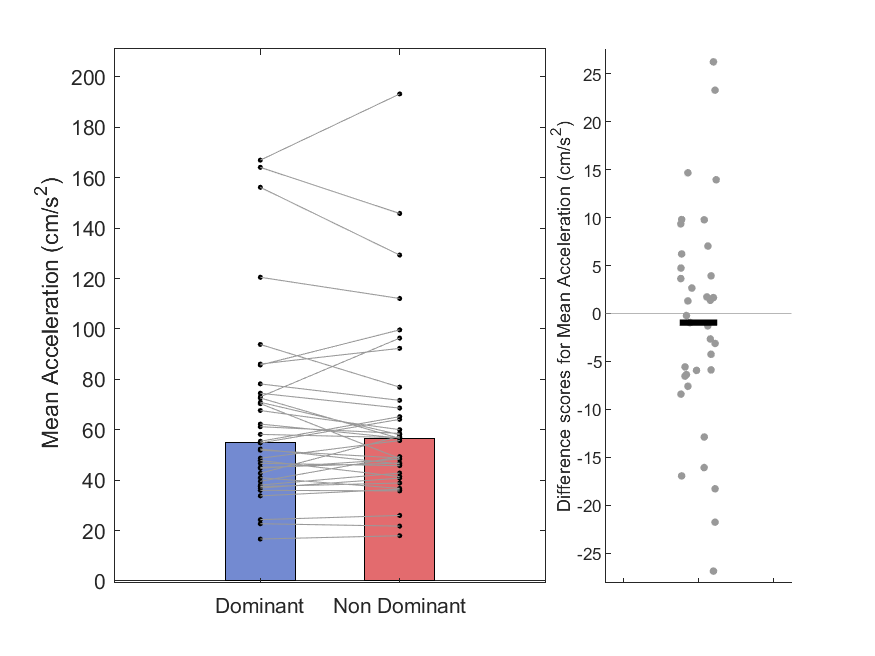


Supplementary Figure 5: Bar and scatter plots with individual matched data points, showing the difference in mean acceleration between the dominant hand compared to the non-dominant hand. Panel B shows the difference scores with the black bar representing the median difference.


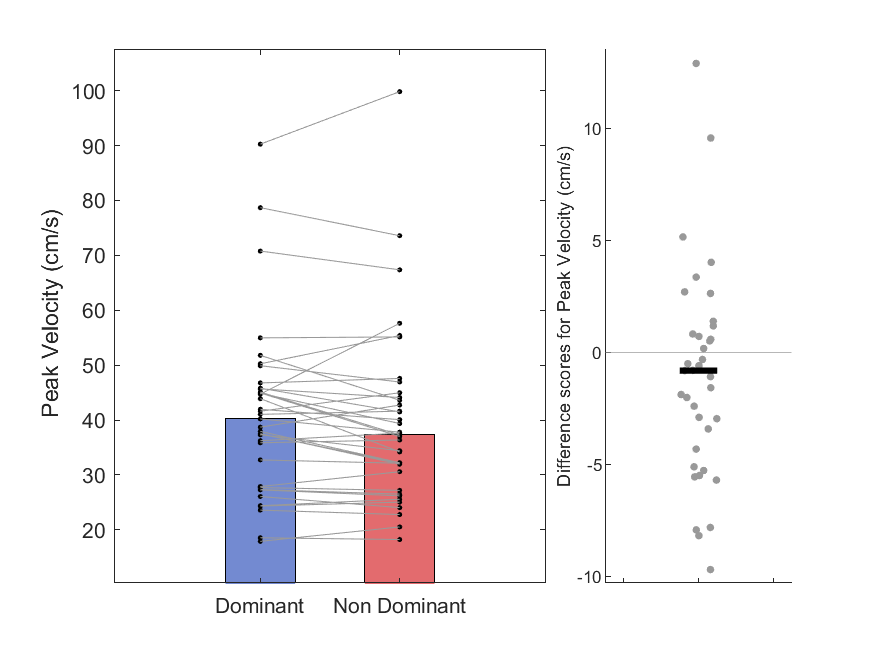


Supplementary Figure 4: Bar and scatter plots with individual matched data points, showing the difference in peak velocity between the dominant hand compared to the non-dominant hand. Panel B shows the difference scores with the black bar representing the median difference.


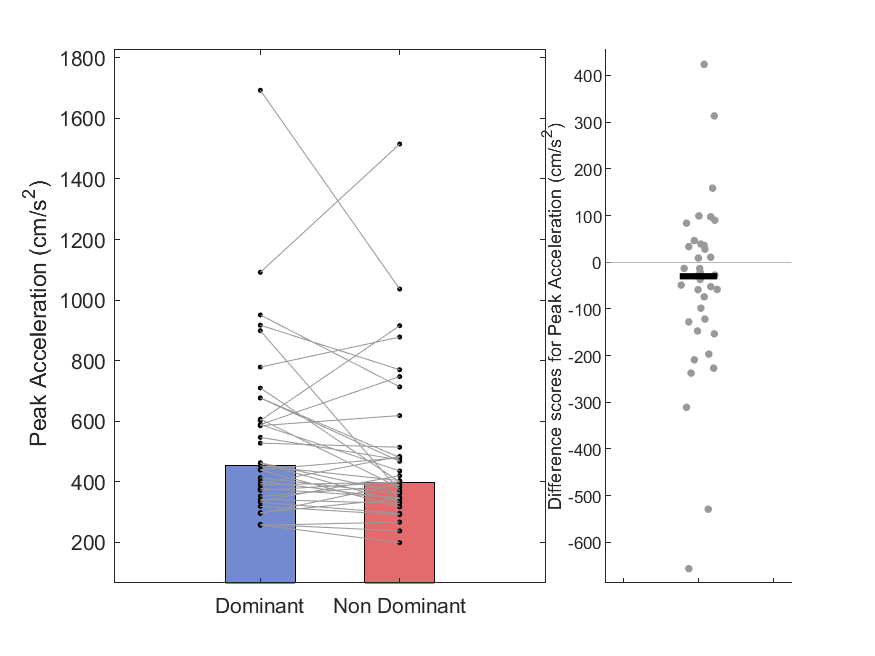


Supplementary Figure 6: Bar and scatter plots with individual matched data points, showing the difference in peak acceleration between the dominant hand compared to the non-dominant hand. Panel B shows the difference scores with the black bar representing the median difference.

## Supplementary Analysis of Potential Outliers


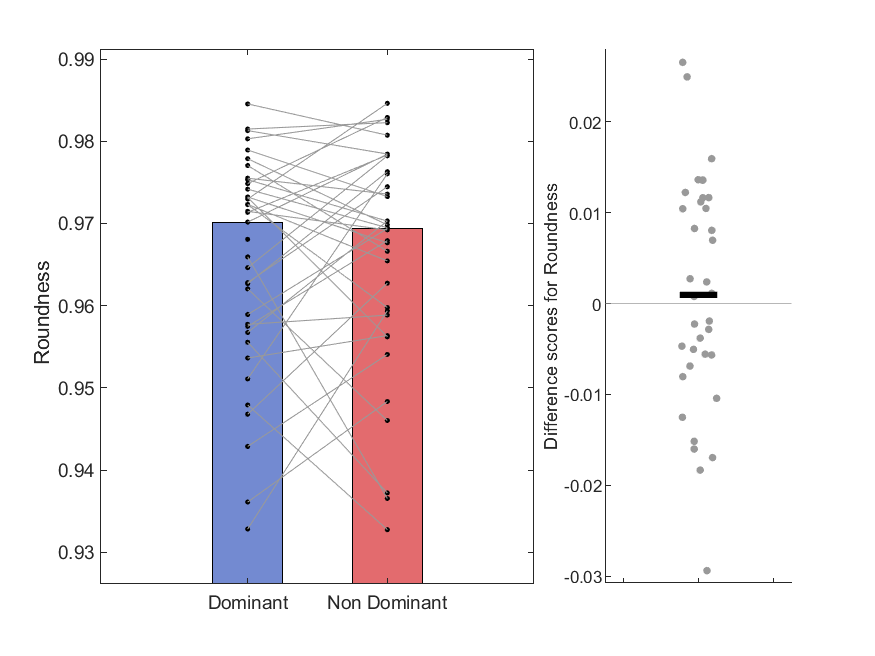
Here we report the results of a supplementary analysis of Circle Roundness and Variation in Area, with potential outlying data points removed. There was no evidence of a difference in circle roundness (Dom *Mdn* = 0.97, *IQR* = 0.02, vs Non-dom *Mdn* = 0.97, *IQR* = 0.02; T = 297, z = -0.57, p = 0.57, r = -0.09); nor in the variation of circle area (Dom *Mdn* = 22.45 cm^2^, *IQR* = 9.50 cm^2^, vs Non-dom *Mdn =* 23.16 cm^2^, *IQR* = 15.50 cm^2^; T = 254, z = -1.24, p = 0.22, r = -0.20) between the two groups.

Supplementary Figure 7: Bar and scatter plots with individual matched data points, showing the difference in roundness between the dominant hand compared to the non-dominant hand, with a potential outlier removed. Panel B shows the difference scores with the black bar representing the median difference.


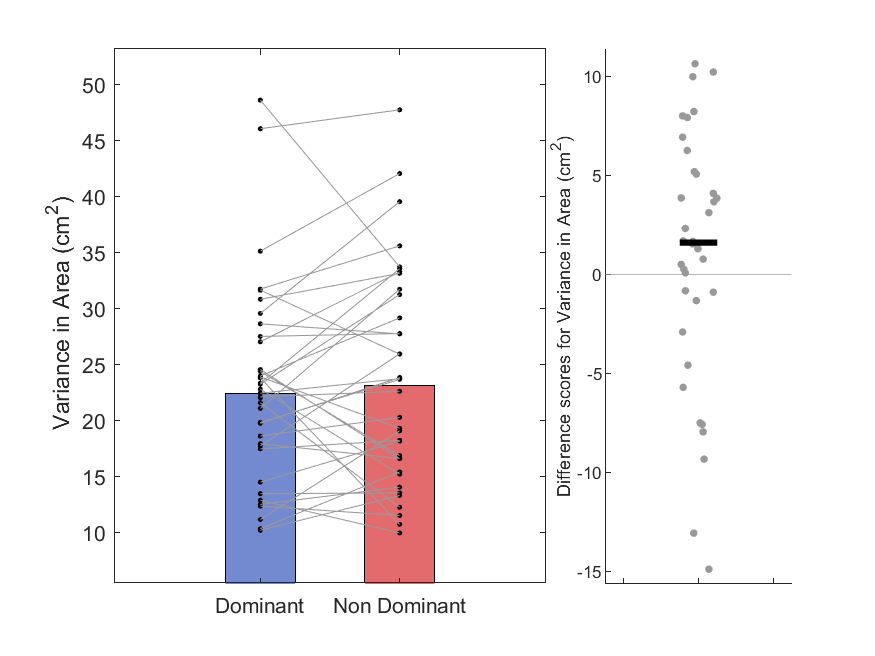


Supplementary Figure 8: Bar and scatter plots with individual matched data points, showing the difference in variance in area between the dominant hand compared to the non-dominant hand, with a potential outlier removed. Panel B shows the difference scores with the black bar representing the median difference.
