# Supplementary material for: Using immersive virtual reality to remotely examine performance differences between dominant and non-dominant hands
Source: Virtual Real. 2023 May 6:1–16. Online ahead of print. doi: 10.1007/s10055-023-00794-z (PMC10162902; doi:10.1007/s10055-023-00794-z)
Supplement: Supplementary file 2 — Supplementary file2 (PDF 118 KB) [file 10055_2023_794_MOESM2_ESM.pdf]

Using Immersive Virtual Reality to remotely examine performance differences between dominant and non-dominant hands.

Virtual Reality

Jack Owen Evans[1], Krasimira Tsaneva-Atanasova[2,3], Gavin Buckingham[1]

[1] Department of Sport and Health Sciences, Richards Building, Magdalen Road, University of Exeter, Exeter, Devon, United Kingdom, EX2 4TA
[2] Department of Mathematics, Living Systems Institute, University of Exeter, Exeter, Devon, United Kingdom, Exeter EX4 4QD
[3] EPSRC Hub for Quantitative Modelling in Healthcare, University of Exeter, Exeter, Devon, United Kingdom, Exeter EX4 4QD

Jack Owen Evans – Corresponding Author
je426@exeter.ac.uk

Transparent Changes

1.1 Sampling

In our original preregistration (see https://doi.org/10.17605/osf.io/t34uq), we identified a target sample size of 90 participants between the ages of 18 and 65. After requests by potential users between the ages of 16 and 18 on social media, we reduced our age restriction to 16+. We also removed our upper age limit of 65. We halted data collection after 7 months having recruited 47 participants. Our original sample size of 90 was based on previous work comparing inter-hand differences. Of the papers we reviewed, most achieved effect sizes of over 0.6 cohen's *d* (Schaffer & Sainburg., 2017; Assi et al., 2016; Carson et al., 1997). Given the differences between our work and previous papers, we originally estimated a conservative cohen's *d* of 0.3 in order to maintain a power of 0.8. Keeping an estimate of 0.3, our adjusted sample of 37 (after data cleaning and drop-outs), results in a prospective power of 0.42. It is worth noting that in practice, however, we observe effect sizes larger than our original estimate (see results). As such, our power is estimated to be higher – achieving 0.68 for our smallest significant effect size.

1.2 Procedure

Originally, we planned for participants to complete both the EHI and the IPQ scales after finishing the main experiment. The experiment was updated so that handedness data was collected

before the main experiment, to prevent missing handedness data in the case of participants stopping the experiment prior to completing the handedness questionnaire.

1.3 Kinematic Measures

1.3.1 Main Analysis

Due to a coding error, we were unable to calculate an originally-planned outcome variable, the Error from target. This would have been calculated by taking the distance (cm) between the outline of the circle drawn by the participant, and the target circle shown to them on the display. We did not collect exact coordinates of the target circle, and so were unable to calculate this metric.

1.3.2 Exploratory Analyses

Following our main analysis and discussions with the research team, we also conducted a number of previously unspecified exploratory analyses to test other potentially interesting metrics. Specifically, we conducted exploratory analyses on path length in the Y axes, mean velocity in the Y axis, variation in velocity in the Y axis, as well as peak velocity, and mean and peak acceleration. These are reported in the Exploratory Analyses section. As the number of right and left handed participants was severely uneven, we did not explore dominant and non-dominant differences between left and right-handed individuals.

1.4 Statistical Analysis Plan

Originally we set our standard alpha-level at 0.05. This has been updated with Bonferroni corrections to adjust for multiple simultaneous comparisons conducted during our main analysis.

1.5 Additional References (for section 1.1 Sampling)

Assi, A., Bakouny, Z., Karam, M., Massaad, A., Skalli, W., & Ghanem, I. (2016). Three-dimensional kinematics of upper limb anatomical movements in asymptomatic adults: Dominant vs. non-dominant. *Human movement science*, *50*, 10-18.

Carson, R. G., Thomas, J., Summers, J. J., Walters, M. R., & Semjen, A. (1997). The dynamics of bimanual circle drawing. *The Quarterly Journal of Experimental Psychology Section A*, *50*(3), 664-683.

Schaffer, J. E., & Sainburg, R. L. (2017). Interlimb differences in coordination of unsupported reaching movements. *Neuroscience*, *350*, 54-64.
