# Supplementary material for: Using immersive virtual reality to remotely examine performance differences between dominant and non-dominant hands
Source: Virtual Real. 2023 May 6:1–16. Online ahead of print. doi: 10.1007/s10055-023-00794-z (PMC10162902; doi:10.1007/s10055-023-00794-z)
Supplement: Supplementary file 3 — Supplementary file3 (DOCX 15 KB) [file 10055_2023_794_MOESM3_ESM.docx]

## **Presence Scores**

Scores from the I-group Presence Questionnaire were averaged across each subscale. Means and standard deviations for each sub-scale are presented in Table 3. The means for each subscale were all above four, indicating that participants felt some sense of presence in the Virtual Environment. In particular, participants indicated a relatively strong sense of physical presence and sense of ‘being there’ in the virtual environment, with average ratings for SP and General above 5.5.

| Table 3: Means and Standard Deviations on the four sub-scales of the I-Group Presence Questionnaire. Scores above 4 indicate a higher sense of presence. | | | |
| --- | --- | --- | --- |
| **Sub Scale** | **Concept Measured** | [**Mean**](#Chapter_2_Litreview) **(Out of 7)** | **Standard Deviation** |
| General Item | Sense of ‘Being There’ | 5.54 | 0.90 |
| Involvement (INV) | Sense of involvement within the environment and awareness of the external space | 4.31 | 1.37 |
| Realism (REAL) | Sense of how realistic the environment feels | 4.13 | 1.17 |
| Spatial Presence (SP) | Sense of being physically present within a space | 5.75 | 0.77 |
